# Design and Pharmaceutical Evaluation of *Piper nigrum* Oil Nanoemulsion by Phase Diagram for Topical Analgesic Applications

**DOI:** 10.5812/ijpr-159933

**Published:** 2025-08-31

**Authors:** Shayan Fallah, Hajar Ashrafi, Mohammad Ali Farboodniay Jahromi, Mohammad Mehdi Zarshenas

**Affiliations:** 1 Medicinal Plants Processing Research Center, Shiraz University of Medical Sciences, Shiraz, Iran; 2 Department of Phytopharmaceuticals (Traditional Pharmacy), Faculty of Pharmacy, Shiraz University of Medical Sciences, Shiraz, Iran; 3 Department of Pharmaceutics, School of Pharmacy, Shiraz University of Medical Sciences, Shiraz, Iran

**Keywords:** Neuropathic Pain, Black Pepper, Topical Formulation

## Abstract

**Background:**

Plant-based pain-relieving formulations have garnered attention in drug discovery, particularly those with a traditional background, often considered essential remedies for communities that rely on plant-based therapies for pain relief.

**Objectives:**

This study aimed to develop and evaluate a semisolid nanoemulsion using *Piper nigrum* L. (black pepper) fruit essential oil, known for its analgesic properties, as the active ingredient.

**Methods:**

A topical nanoemulsion was prepared under standard conditions using black pepper essential oil, emulsifiers, and excipients. Various combinations of Span 80 and Tween 80 were screened to achieve the desired hydrophilic-lipophilic balance (HLB) value. Among the several formulations prepared, a nanoemulsion sample was selected for detailed analytical and pharmaceutical evaluations. Gas chromatography with flame ionization detection (GC/FID) was used to identify and quantify major chemical constituents of the essential oil and the formulated nanoemulsion.

**Results:**

An optimized combination of Span 80 and Tween 80, with an HLB of 11, contributed significantly to the physical stability of the formulations. A concentration of 2.7% *P. nigrum* essential oil in the nanoemulsion containing 3793.33 ± 222.75 µg/mL of caryophyllene, the major bioactive monoterpene, rendered a stable and acceptable nanoemulsion product. The nanoemulsion formulation ST42 was declared to have a viscosity of 1.8 MPa.s, ST mix (Span 80/Tween 80, 1.5:2.5), and a ratio of surfactant/essential oil (40:60), demonstrated optimal consistency and physical stability. The zeta potential (Z) of the optimized formulation ST42 was found to be close to neutral (-8.12 ± 2.0 mV), minimizing potential tissue irritation. The nanoemulsion was ultimately validated using a modified homogenization technique to improve droplet size, stability, and rheological characteristics.

**Conclusions:**

The formulated black pepper nanoemulsion successfully passed key pharmaceutical quality tests, indicating its potential as a natural topical pain-relieving agent. Further in vivo studies and subsequent clinical trials may lead to the development of a plant-based product for managing neuropathic pain.

## 1. Background

Pain, often resulting from conditions like tumors, surgery, or trauma (1), initially serves as a warning signal and a protective tool. However, when severe, it can lead to complications such as anxiety, palpitations, nausea, and a reduced quality of life (2). Conventional treatments for pain and inflammation commonly include aspirin, acetaminophen, NSAIDs (ibuprofen and naproxen), corticosteroids, and opioids (3). The main disadvantage of these drugs is the well-documented toxicity linked to the inhibition of cyclooxygenase and the decline in prostaglandin levels, leading to renal toxicity and the development of gastrointestinal and cardiovascular side effects (4). Long-term treatment with systemic corticosteroids may cause severe unwanted reactions, such as high blood pressure, diabetes, gastric ulcers, osteoporosis, and some have the potential for addiction (5).

In Traditional Persian Medicine, essential oils and plant-based remedies are widely used for pain relief (6). Essential oils contain mono- and sesquiterpenoids that exhibit analgesic effects by inhibiting the peripheral and central nervous systems, thereby blocking the transmission of pain signals (7). Black pepper (*Piper nigrum* L.), a member of the Piperaceae family, has been traditionally used in Persian medicine for various ailments, including pain and inflammation, and was selected for this study. Studies have shown that black pepper essential oil reduces pain intensity. The α-Pinene, a marker constituent of the oil, has exhibited anti-inflammatory, anti-osteoarthritic, and antinociceptive properties in animal models (8, 9). The α-Pinene has demonstrated strong anti-inflammatory properties in human chondrocytes, as well as anti-osteoarthritic effects and potent inhibition of IL-1β-induced inflammatory pathways (9).

A topical nanoemulsion form was selected as the route of administration to achieve the most efficient therapeutic efficacy, including superior dispersibility, localized therapeutic action, reduced systemic side effects, higher effectiveness, and improved patient compliance (10). Nanoemulsions enhance skin permeability and controlled drug release, exhibiting exceptional physicochemical characteristics that make them ideal for topical use (11). Their small droplet size increases the solubility and skin absorption of lipophilic drugs, improving bioavailability and therapeutic effectiveness (11). Thus, they offer a promising alternative to conventional drug delivery systems.

## 2. Objectives

This study aimed to develop a nanoemulsion as a topical pain-relief formulation, based on black pepper essential oil, targeting enhanced bioavailability, solubility, and skin penetration to improve therapeutic efficacy and patient acceptance, in line with traditional Persian medicine and pharmaceutical principles.

## 3. Methods

### 3.1. Plant Material

Black pepper (*P. nigrum* L.) fruits were procured from a certified market in Shiraz and authenticated by the taxonomist of the Department of Traditional Pharmacy, Faculty of Pharmacy, Shiraz University of Medical Sciences.

### 3.2. Essential Oil Extraction

Dried fruits were procured from an authorized herbal store in Shiraz, milled, and essential oil was extracted for 5 hours using a Clevenger-type apparatus. The extracted oil was then stored at -20°C before undergoing GC/MS analysis.

### 3.3. Identification of Essential Oil Components-GC/MS Analysis

Identification of volatile components in black pepper essential oil was performed using an earlier reported standard GC/MS procedure (12). The Kovats Index of each volatile component was calculated and compared with data from the nl7 library, Adams' book of essential oil components (13), NIST, and Pherobase databases (14, 15), as well as relevant reported data.

### 3.4. Identification of Volatile Constituents of the Nanoemulsion-GC/MS Analysis

The volatile oil from the nanoemulsion was extracted by hydrodistillation using a Clevenger apparatus, then diluted to 47,000 µg/L for GC/MS analysis to identify its chemical components.

### 3.5. Preparation of Oil-in-Water Emulsion

An oil-in-water emulsion was prepared by mixing heated black pepper essential oil with a surfactant-water mixture at 40°C, followed by homogenization at 13,000 rpm for 10 minutes (16). Three surfactant types were used: Tween 80, Span 80, and a Tween 80 - Span 80 mixture adjusted to a hydrophilic-lipophilic balance (HLB) of ~11 for optimal stability (17). The HLB was used to determine the optimal ratio of Tween 80 and Span 80 for a stable oil-in-water emulsion. A suitable HLB value for preparing an oil-in-water emulsion has been reported, ranging from 8 to 16 (17). Various emulsions prepared were analyzed to determine the optimal ratios of ingredients in the final formulation. A ternary phase diagram relevant to stability, transparency, single-phase uniformity, and droplet size was drawn (18).

### 3.6. Standardization of Nanoemulsion-Gas Chromatography with Flame Ionization Detection

Caryophyllene content in the nanoemulsion was quantified using gas chromatography with flame ionization detection (GC/FID) (Agilent 7890A/5975C) after GC/MS identification of volatile components. Analysis was performed using an HP-5 column with a temperature gradient from 60°C to 280°C (6°C/min), with nitrogen as the carrier gas. A 47 mg/L solution of the volatile fraction was analyzed alongside standard caryophyllene dilutions (1700 - 27200 μg/mL). Injections (1 μL, split 1:50) were performed in triplicate, and quantification was based on calibration curves. Accuracy was validated via coefficient of variation and relative standard deviation (SD) (19).

### 3.7. Evaluation of the Pharmaceutical Characteristics of the Emulsions

The nanoemulsion's organoleptic properties, pH, conductivity, and viscosity were examined in triplicate on days 1, 2, 3, 8, 15, and 30 after production, and the viscosity was measured (20). Physical stability and phase separation were monitored at room and refrigerated temperatures. The stability test was performed upon centrifugation using a Z200A centrifuge (Hermle LaborTechnik GmbH) at a speed of 3000 rpm at different intervals between 5 and 30 minutes (20). To assess emulsion stability, the droplet size and zeta potential (Z) were evaluated using a Shimadzu Particle Size Analyzer (SLAD-2101, Japan) on days 0, 1, 3, 6, 7, and 15, with three reproducible measurements (17, 21).

### 3.8. Statistical Analysis

All experiments were performed in triplicate over a minimum of three independent trials. Results are displayed as mean ± SD. Statistical significance was assessed through one-way ANOVA, and a P-value < 0.01 was considered significant. The data analysis was performed using GraphPad Prism 6.00 software.

## 4. Results

### 4.1. Identification of Chemical Constituents of Essential Oil

**Figure 1.**
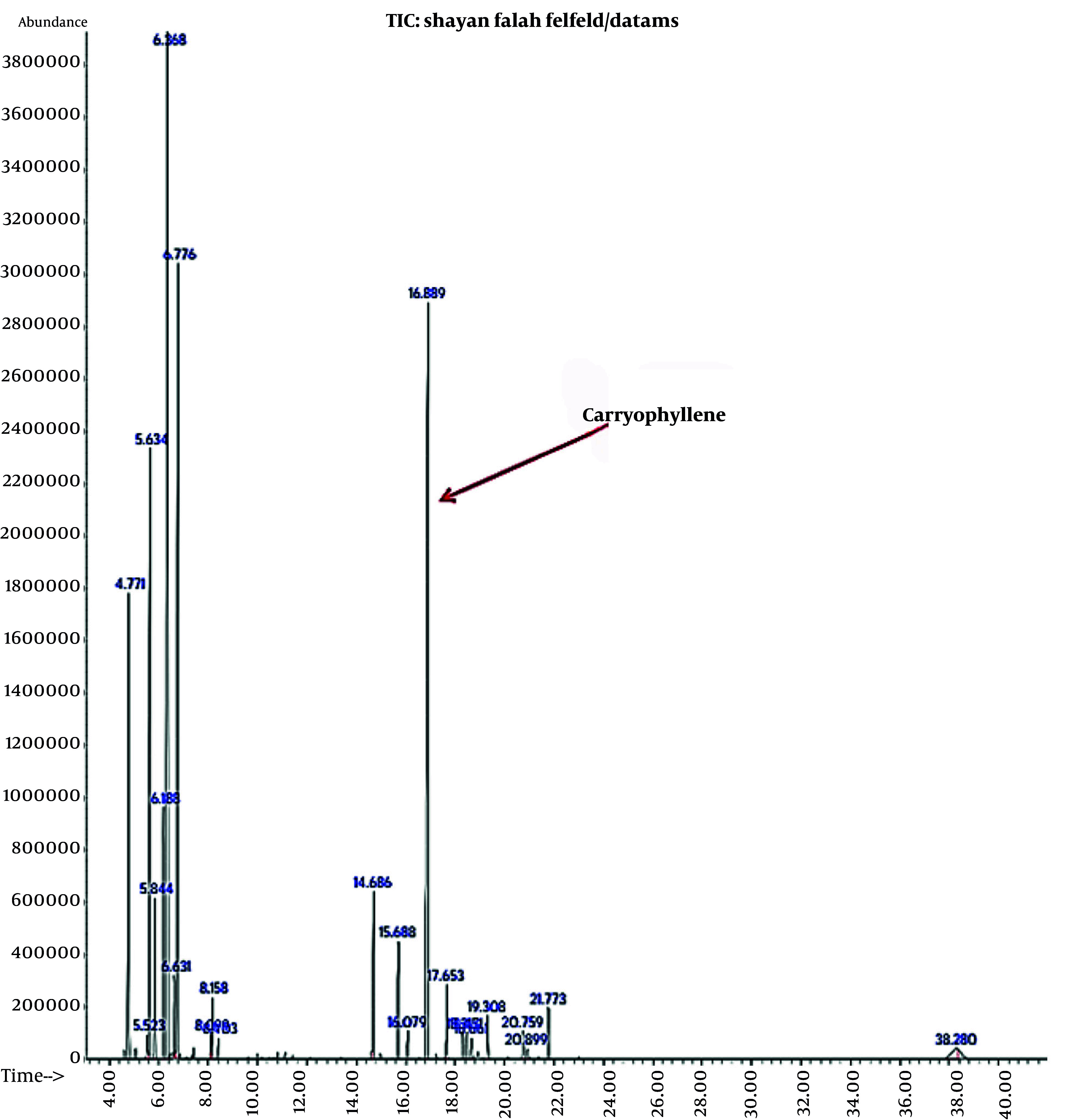
GC Chromatogram of black pepper seed essential oil

The essential oil yield from black pepper seeds was 0.7%, with 16 compounds identified via GC/MS analysis, as shown in Figure 1. 

The GC/MS analysis results of black pepper fruit essential oil are presented in Table 1, which indicates that the predominant constituents of the oil are δ-3-Carene (22.97%) and caryophyllene (21.33%).

**Table 1.** Volatile Components of Black Pepper Fruit Essential Oil

Components	Area (%)	RICal	Ref
**α-Pinene**	7.03	936	936
**Sabinene**	0.45	975	975
**β-Pinene**	9.86	981	981
**β-Myrcene**	2.43	992	992
**Phellandrene**	4.89	1008	1008
**δ-3-Carene**	22.97	1015	1015
**Limonene**	16.42	1032	1032
**Isoterpinolene**	0.36	1088	1088
**Linalool**	0.30	1100	1100
**δ-Elemene**	2.79	1341	1341
**α-Copaene**	1.93	1380	1380
**trans-Caryophyllene**	21.33	1428	1428
**α-Humulene**	1.26	1459	1459
**Germacrene D**	0.46	1486	1486
**α-Selinene**	0.42	1500	1498
**δ-Cadinene**	0.72	1547	1543
**Total identified compounds (%)**	95.08		
**Monoterpenes**	66.17		
**Sesquiterpenes**	28.91		

Abbreviations: RICal, Calculated Retention Index; RIRef, Reported Retention Index.

### 4.2. Formulation of the Nanoemulsion

**Figure 2.**
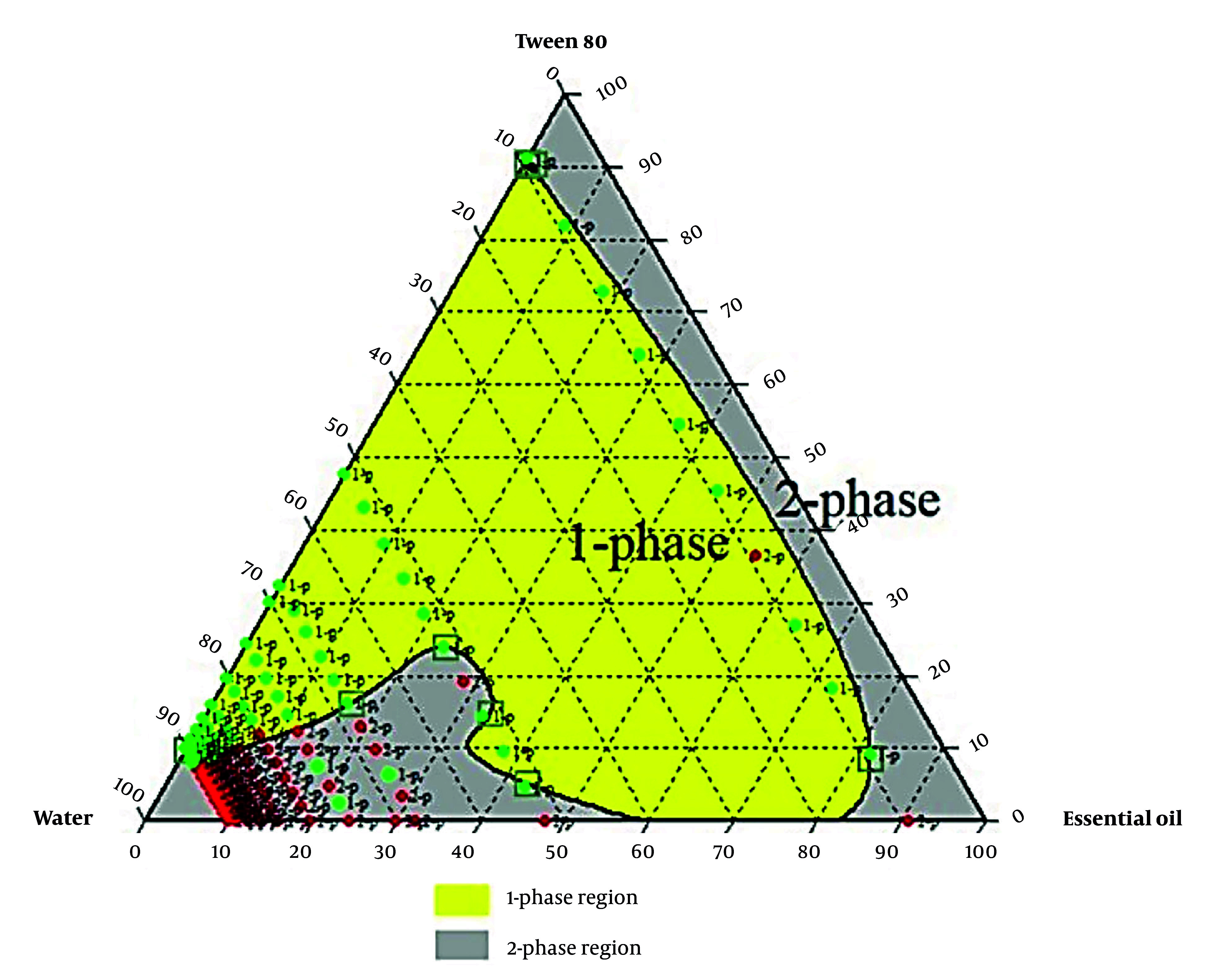
Ternary phase diagram for the emulsions with Tween 80

**Figure 3.**
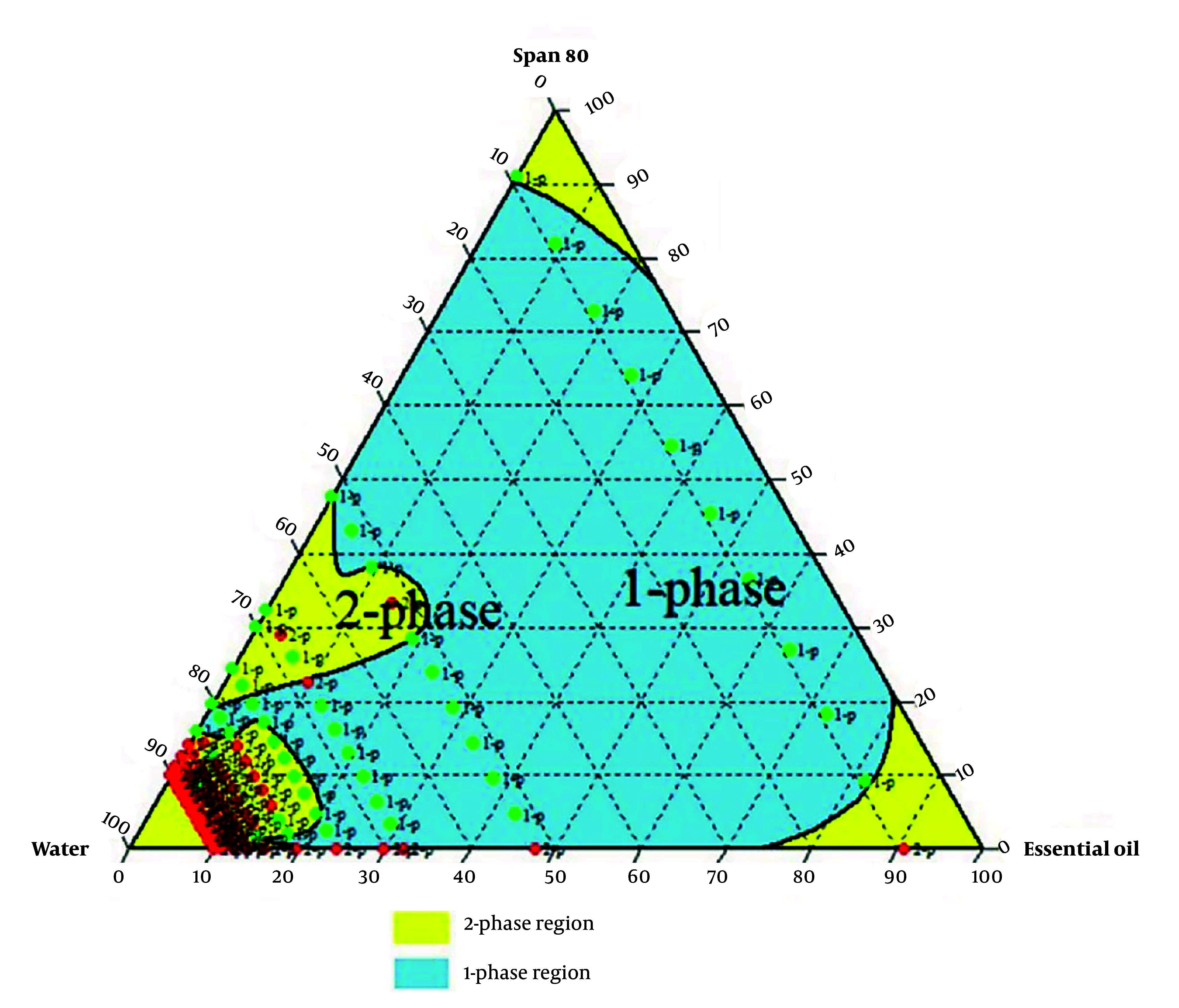
Ternary phase diagram for the emulsions with Span 80

**Figure 4.**
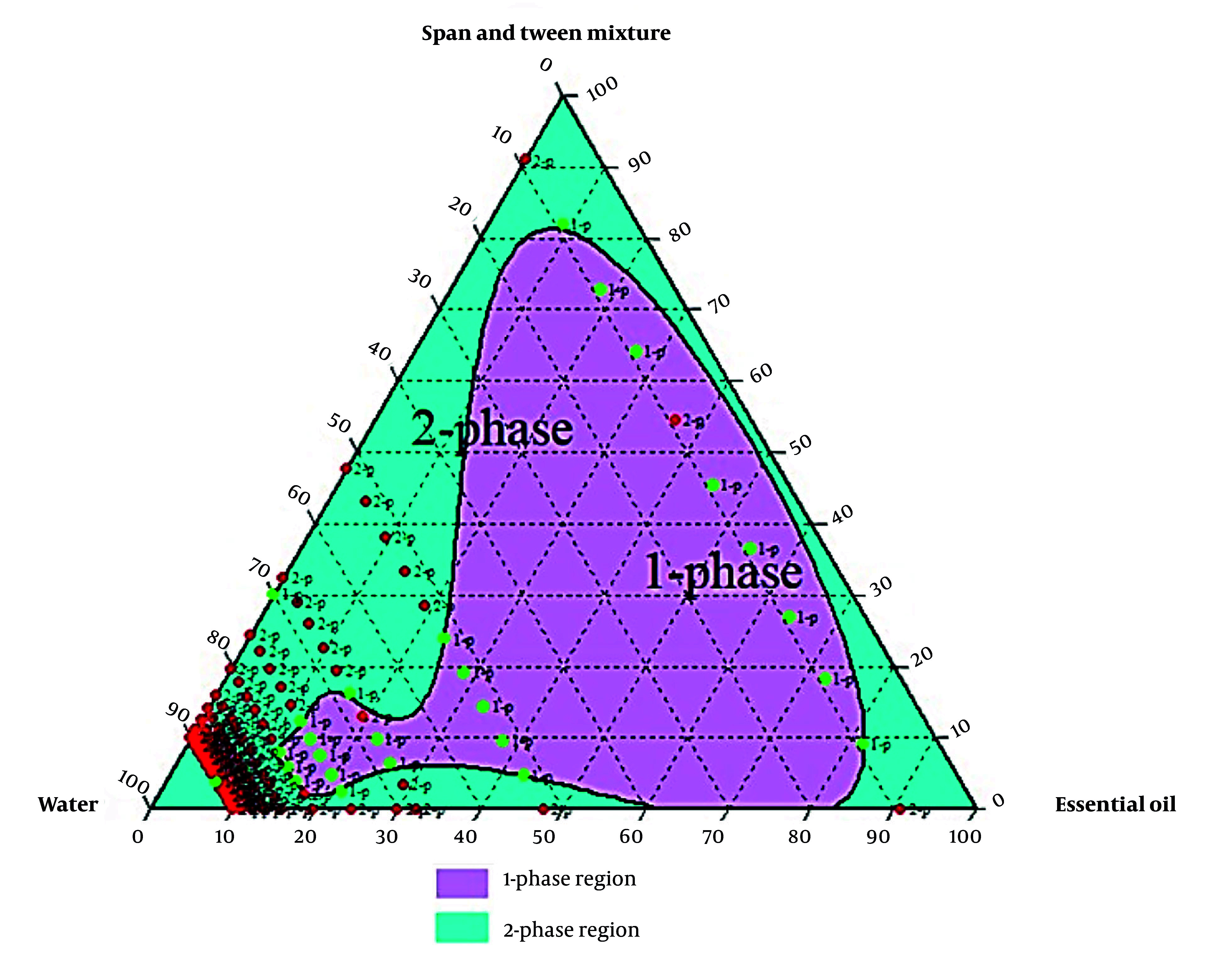
Ternary phase diagram for the emulsions with Tween and Span mixture

Formulations with varying essential oils, surfactants (Tween 80, Span 80, or their mix at HLB 11), and water were tested. Emulsion states were analyzed, and ternary phase diagrams of 330 samples (Chemix v11.5) were generated, showing monophasic (green) and biphasic (red) points, as illustrated in Figures 2 - 4. 

Among all 330 formulations prepared, the monophasic formulations with smaller droplet sizes were selected for further analysis (Table 2). 

**Table 2.** Monophasic Formulations in the Middle of the Monophasic Zone of the Diagram ^a^

Formulations	Ess. Oil/Surf.	H_2_O (mL)	Ess. Oil (%)	Droplet Size µm (N)	Droplet Size µm (V)	Homogeneity	Approval	Disapproval (Reasons)
**T21**	8:2 T	1	7.2	0.55	0.96	+	×	HEOC
**T22**	8:2 T	2	3.8	0.72	1.2	+		-
**T51**	5:5 T	1	4.5	0.44	1.4	+	×	HEOC
**T61**	4:6 T	1	3.64	1.00	4.9	+	×	Droplet size
**T71**	3:7 T	1	2.69	1.00	5.00	+	×	Droplet size
**S11**	9:1 S	1	8.3	0.58	2.00	+	×	HEOC
**S12**	9:1 S	2	4.3	0.66	2.30	+	×	HEOC
**S13**	9:1 S	3	3.0	0.60	4.20	+	×	Droplet size
**S21**	8:2 S	1	7.27	0.49	0.80	+	×	HEOC
**S22**	8:2 S	2	3.8	0.84	1.40	-+	×	Homogeneity
**S31**	7:3 S	1	6.4	0.43	12.10	-	×	Homogeneity
**S32**	7:3 S	2	3.3	0.60	18.11	-	×	Homogeneity
**S41**	6:4 S	1	5.4	0.98	8.40	-	×	Homogeneity
**S42**	6:4 S	2	2.9	0.45	7.90	-+	×	Homogeneity
**S51**	5:5 S	1	4.5	0.35	0.88	+	×	HEOC
**S61**	4:6 S	1	3.6	0.57	1.30	+		-
**S71**	3:7 S	1	2.7	0.53	1.00	+		-
**ST21**	8:2 M	1	7.27	0.73	1.60	+	×	HEOC
**ST22**	8:2 M	2	3.8	0.75	1.80	+	×	Droplet size
**ST31**	7:3 M	1	6.4	0.38	1.10	+	×	HEOC
**ST32**	7:3 M	2	3.3	0.50	1.90	+	×	Droplet size
**ST41**	6:4 M	1	5.5	0.13	0.74	+	×	HEOC
**ST42**	6:4 M	2	2.8	0.51	1.40	+		-
**ST51**	5:5 M	1	4.5	0.56	2.20	+	×	HEOC
**ST71**	3:7 M	1	2.7	0.67	0.74	+		-

Abbreviation: HEOC, high essential oil content.

^a^ T: Tween-80; S: Span-80; M: Mixed surfactant; part size: Droplet; N: Number-based; V: Volume-based.

Formulations with smaller droplet sizes (based on both number and volume) and more suitable surfactant combinations were selected for further evaluation (Table 3). 

**Table 3.** Further Detailed Pharmaceutical Evaluation of the Selected Formulations

Formulations	Sodium Metabisulfite (mg)	Methylparaben (mg)	Propylparaben (mg)	Water (mL)	Surfactant (µL)	Ess. Oil (µL)
**T22**	2.1	0.63	0.21	2.0	20	80
**S61**	1.1	0.33	0.11	1.0	60	40
**S71**	1.10	0.33	0.21	1.0	70	30
**ST42**	2.1	0.63	0.21	2.0	40 (T/S 25:15)	60
**ST71**	1.1	0.33	0.11	10	70 (T/S 43.7:26.3)	30

Five formulations (T22, S61, S71, ST42, and ST71) were selected for pharmaceutical analysis after meeting initial specifications (Table 3). These formulations were scaled up and evaluated, with results shown in Table 4. Of the five, three (T22, ST42, and ST71) demonstrated properties closest to standard values and were selected for further analysis.

**Table 4.** Pharmaceutical Characteristic Tests of Selected Formulations ^a^

Formulations	pH				Conductivity (mV)				Centrifuge		Average Potential (Z)
	Day 1	Day 3	Day 7	Day 15	Day 1	Day 3	Day 7	Day 15	5.0 min	30 min	
**T22**	3.17 ± 0.09	3.21 ± 0.01	3.21 ± 0.02	3.25 ± 0.03	181.1 ± 1.1	188.4 ± 1.27	191.8 ± 0.8	191.5 ± 0.7	BP	BP	-2.11 ± 4.0
**ST42**	3.30 ± 0.03	3.17 ± 0.02	2.97 ± 0.02	2.99 ± 0.02	186.7 ± 1.1	197.4 ± 0.8	209.4 ± 0.6	208.1 ± 0.8	ST	BP	-8.12 ± 2.0

Abbreviation: ST, standard.

^a^ Values are expressed as mean ± standard deviation (SD).

### 4.3. Evaluation of the Pharmaceutical Properties of the Emulsion

The organoleptic properties of the selected formulations, T22, ST42, and ST71, were monitored over time, showing no significant changes in color or appearance during the first 14 days. However, on day 15, the T22 sample exhibited a slight increase in droplet size. By day 30, the physical appearance of the two stable samples (ST42 and ST71) remained unchanged, whereas T22 showed a further increase in droplet size and early signs of phase separation.

### 4.4. The Nanoemulsion's pH

The acidity of skincare formulations is crucial for maintaining skin integrity, as pH changes can affect barrier function and lead to various skin disorders. The pH of formulation ST71 was evaluated and found to be 3.64 ± 0.13, a level considered acceptable for topical use (Table 4). 

### 4.5. Conductivity Test

The nanoemulsion's electric conductivity was measured to assess ion content and formulation stability. A sharp change in conductivity indicates a possible disorder in the formulation components. A value of 196.56 ± 6 μS/cm^2^ was recorded over 30 days, indicating stable conductivity. According to USP monograph criteria, the formulation qualified as an oil-in-water emulsion (22). The centrifugation testing, which simulates aging, was used to assess the emulsion's long-term stability (17). At 3000 rpm for 5 and 30 minutes, no phase separation was observed in formulation ST71. As shown in Table 4, the zeta potential was close to neutral, indicating low tissue irritation, which supports findings from similar studies (21).

### 4.6. Components of the Final Nanoemulsion

Table 5 presents the proportions and weight percentages of the components in the nanoemulsion formulations. These formulations were then examined for compatibility with standard pharmaceutical data.

**Table 5.** The Components' Proportions of the Final Nanoemulsions

Formulation	Span 80 (μL)	Tween 80 (μL)	Methyl Paraben (w/w %)	Propyl Paraben (w/w %)	Sodium Sulfite (w/w %)	Essential Oil (μL)	Water (μL)
**T22**	0	20	0.03	0.01	0.1	80	2
**ST42**	15	25	0.03	0.01	0.1	60	2
**ST71**	26.3	43.7	0.03	0.01	0.1	30	1

### 4.7. Evaluation of the Rheological Properties

The nanoemulsion's viscosity behavior was studied and analyzed using an Ostwald viscometer, based on flow resistance (17, 23). The times recorded were applied to the relevant formula (Equation 1) to calculate viscosity, and the results were expressed in seconds (Table 6). 


Equation 1: $$\frac{\eta^{1}}{\eta^{2}}=\frac{t_{1}d_{1}}{t_{2}d_{2}}$$

The density of the samples was determined by measuring the weight of 1 mL of the sample, as shown in Equation 1.

- T22: 1.18

- ST42: 1.25

- ST71: 1.29

- For T22, considering that the viscosity of water at 25˚C is 1.0: 1/(Formulation Viscosity) = (10.27 × 1.0)/(10.47 × 1.18) so viscosity = 1.20 MPa.s.

- For ST42, considering that the viscosity of water at 25˚C is 1.0: 1/(Formulation Viscosity) = (10.27 × 1.0)/(11.23 × 1.25) so viscosity = 1.37 MPa.s.

- For ST71, considering that the viscosity of water at 25˚C is 1.0: 1/(Formulation Viscosity) = (10.27 × 1.0) / (1.29 × 14.37) so viscosity = 1.8 MPa.s.

**Table 6.** Determination of the Time Recorded for Each Formulation

Sample Volume (mL)	Emulsion Type (s)			
	T22	ST42	ST71	Water
**7.0**	9.91 ± 0.04	10.84 ± 0.05	14.21 ± 0.1	9.89 ± 0.06
**10.0**	10.47 ± 0.02	11.23 ± 0.01	14.37 ± 0.05	10.27 ± 0.05

^a^ Values are expressed as mean ± standard deviation (SD).

### 4.8. Evaluation of Physical Stability

The nanoemulsion's physical stability was assessed at various time intervals, including 0, 1, 3, 6, 7, 15, and 30 days at room temperature and 4°C (11). No significant changes in appearance or stability were observed, indicating that formulation ST71 maintained optimal consistency and stability.

### 4.9. Determination of Droplet Size

**Figure 5.**
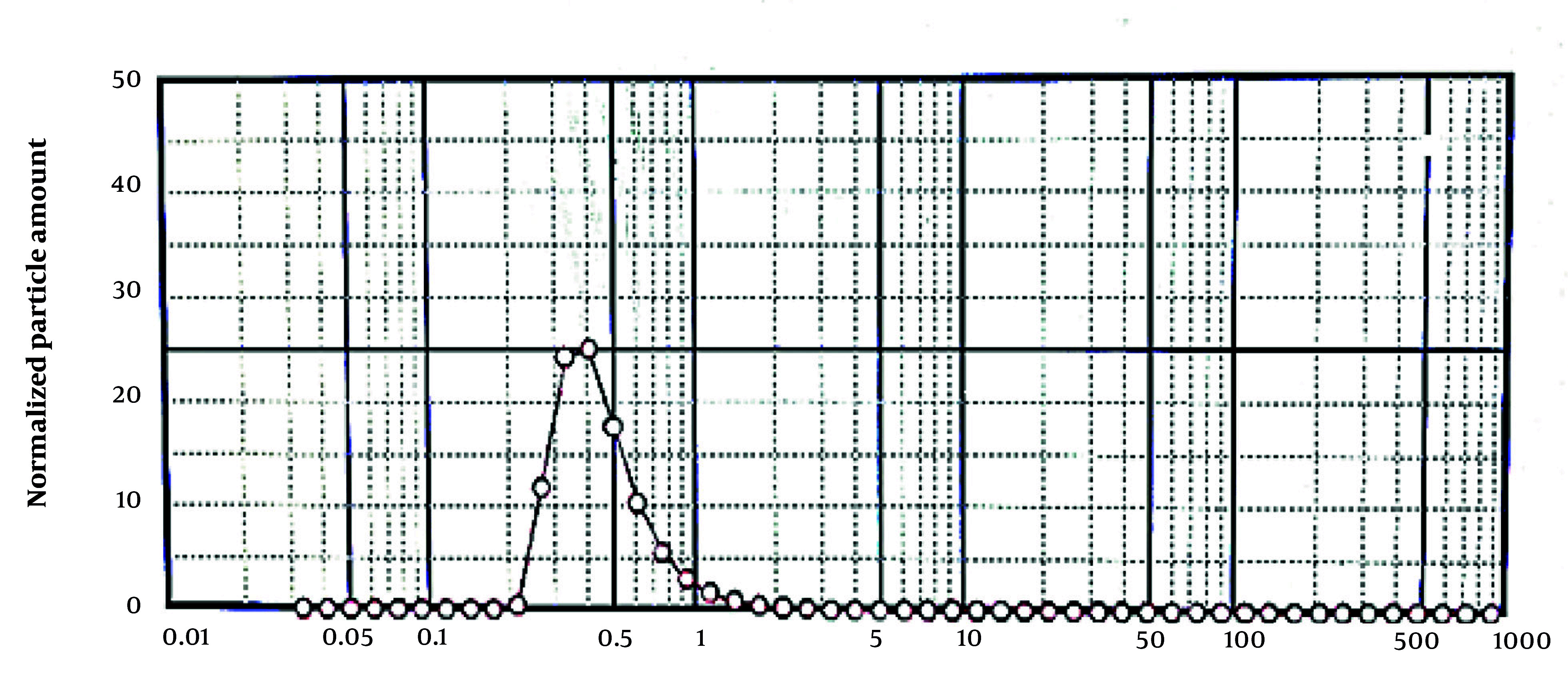
Determination of droplet size of the nanoemulsion formulation

Figure 5 shows that the formulated nanoemulsion droplets are in the nanometer range, achieved through homogenization at 13,000 rpm. Droplet size is a critical factor influencing emulsion stability (17).

### 4.10. Caryophyllene Content of the Final Nanoemulsion Product-Gas Chromatography with Flame Ionization Detection

The density of 1 mL of the nanoemulsion's volatile fraction was approximately 0.80 g/cm^3^. The caryophyllene content of this portion (1 mL) was measured as 3793.33 ± 222.75 µg/mL (Table 7). A 2.7% concentration of *P. nigrum* fruit essential oil was used to develop a stable and acceptable nanoemulsion.

**Table 7.** Caryophyllene Content of the Volatile Fraction of the Nanoemulsion

No.	Area (%)	Con. (μg/mL)	Mean ± SD (μg/mL)	LOD (μg/mL)	LOQ (μg/mL)
**1**	1627.48	3536.19	3793.33 ± 222.75	60	180
**2**	1830.32	3916.50			
**3**	1836.07	3927.30			

## 5. Discussion

Research prioritizes finding safe alternatives to NSAIDs and opiates, and among natural alternative agents, *Piper* species offers a hopeful and optimistic outlook for the future of pain management (24).* Piper nigrum* fruits are deeply rooted in Persian, Ayurvedic, and Unani medicine for fever, pain, and inflammation. This traditional context adds a logical sense of connection and continuity to the present research (25). Considering the crucial need for new pain-relieving drugs, particularly those of natural origin, this study successfully developed a topical nanoemulsion containing black pepper fruit essential oil, a widely accepted analgesic agent, as noted in Qarabadin's text, an authentic traditional medicine source. Using the Chemix school software to draw triangle diagrams facilitated the process of creating the formulations. Specific ratios of Span 80 and Tween 80 enabled the formation of a stable oil-in-water emulsion. Optimal stability of the nanoemulsion was achieved with a 2.7% essential oil content, proper surfactant ratio, and homogenization parameters within the single-phase region of the ternary phase diagram (Figures 2 - 4). Several single-phase formulations were compared for oil content, droplet size, and consistency. The zeta potential of the formulated nanoemulsion was found to be close to neutral, which reflects the particle surface charge, emulsion stability, and cellular absorption by influencing the electrostatic interactions of moving droplets. The resulting nano-sized emulsions revealed improved stability and reduced tissue irritation, as confirmed by similar studies (17).

The GC/FID proved effective for identifying and quantifying key oil components. Results indicated that black pepper fruit essential oil comprises monoterpenes (70%) and sesquiterpenes (30%), which align with previous studies (26). The oil's marker components were δ-3-carene (23%), caryophyllene (21.3%), and limonene (16.4%). β-Caryophyllene acts as a cannabinoid receptor 2 agonist, functioning as a pain-relief agent (27). δ-3-Carene and caryophyllene also exhibit anti-inflammatory activity (IC_50_ 0.0008 - 0.02%), reducing IL-6 secretion by up to 60% at a concentration of 0.01% (28). Earlier research also reported that δ-3-Carene and β-caryophyllene, isolated from Qianghuo volatile oil — a currently used Chinese herbal anti-inflammatory drug — demonstrated in vitro inhibition and in vivo anti-inflammatory activity, and were found to be potent inhibitors of COX-2, with IC_50_ values of 13.5 and 10.1 μM, respectively (29). Thus, the notable pain-relieving properties of black pepper oil may be attributed to these terpenes.

In the present study, the five key components of *P. nigrum* fruits essential oil, identified by GC/MS analysis, were δ-3-carene, trans-caryophyllene, limonene, β-pinene, and α-pinene — all of which are known for their anti-inflammatory and analgesic properties. The α-Pinene, δ-3-carene, and limonene, the abundant components of *Angelica archangelica* essential oil, induced significant apoptosis and necrosis in human histiocytic lymphoma cells (30). δ-3-Carene, a bicyclic monoterpene, was the most abundant compound in both black pepper essential oil and the optimized nanoemulsion formulation, significantly contributing to its anti-inflammatory activity. This compound contributed to the anti-inflammatory effects of the essential oil of Bupleurum gibraltaricum on carrageenan-induced pedal edema in rats (31).

The β-Caryophyllene, a bicyclic sesquiterpene and non-toxic compound with an LD_50_ greater than 5000 mg/kg, reduces inflammation by inhibiting inflammatory mediators and regulating cell proliferation (32). Limonene alleviates conditions like colitis and pneumonia by modulating inflammatory signaling (33). The α- and β-Pinene exhibit anti-inflammatory and anti-apoptotic effects by suppressing related cytokines and pro-apoptotic genes (34).

### 5.1. Conclusions

The nanoemulsion fabricated in the present research demonstrates the capacity to overcome the limitations associated with conventional topical drug delivery systems. These efforts may ultimately lead to an improved mode of administration of a pain-relieving product based on *P. nigrum* essential oil components, potentially enhancing patient compliance and reducing side effects. The nanoemulsion has successfully passed the necessary physicochemical evaluation tests, instilling confidence in its potential. However, it must undergo further comprehensive in vivo assessments and standard pharmaceutical quality assays before its use as a topical formulation to manage pain and inflammation.

## Data Availability

The data procured in the present study is accessible at the request, if necessary, during the submission or after publication.

## References

[1] Apkarian AV, Bushnell MC, Treede RD, Zubieta JK (2005). Human brain mechanisms of pain perception and regulation in health and disease.. Eur J Pain..

[2] Schmidt R, Schmelz M, Forster C, Ringkamp M, Torebjork E, Handwerker H (1995). Novel classes of responsive and unresponsive C nociceptors in human skin.. J Neurosci..

[3] Fields HL (2011). The doctor's dilemma: opiate analgesics and chronic pain.. Neuron..

[4] Chan HCS, McCarthy D, Li J, Palczewski K, Yuan S (2017). Designing Safer Analgesics via mu-Opioid Receptor Pathways.. Trends Pharmacol Sci..

[5] Rice JB, White AG, Scarpati LM, Wan G, Nelson WW (2017). Long-term Systemic Corticosteroid Exposure: A Systematic Literature Review.. Clin Ther..

[6] Hamedi A, Zarshenas MM, Sohrabpour M, Zargaran A (2013). Herbal medicinal oils in traditional Persian medicine.. Pharm Biol..

[7] Perri F, Coricello A, Adams JD (2020). Monoterpenoids: The Next Frontier in the Treatment of Chronic Pain?. Multidisciplinary Sci J..

[8] R.Costa, J.Machado, C.Abreu (2016). Evaluation of Analgesic Properties of Piper Nigrum Essential Oil: a Randomized, Double-blind, Placebo-controlled Study.. World J Traditional Chinese Med..

[9] Rufino AT, Ribeiro M, Judas F, Salgueiro L, Lopes MC, Cavaleiro C (2014). Anti-inflammatory and chondroprotective activity of (+)-alpha-pinene: structural and enantiomeric selectivity.. J Nat Prod..

[10] Zhao L, Chen J, Bai B, Song G, Zhang J, Yu H (2023). Topical drug delivery strategies for enhancing drug effectiveness by skin barriers, drug delivery systems and individualized dosing.. Front Pharmacol..

[11] Rai VK, Mishra N, Yadav KS, Yadav NP (2018). Nanoemulsion as pharmaceutical carrier for dermal and transdermal drug delivery: Formulation development, stability issues, basic considerations and applications.. J Control Release..

[12] Jahromi MAF, Etemadfard H, Zebarjad Z (2016). Chemical Characterization and Antimicrobial Activity of Essential oil from the Leaves of Bienertia cycloptera.. Chem Natural Compounds..

[13] Adams RP (2007). Identification of Essential Oil Components by Gas Chromatography/mass Spectrometry..

[14] Heller SR, Milne GWA (2001). EPA/NIH Mass Spectral Data Base..

[15] The Pherobase: the database of pheromones and semiochemicals (2025). KovatsIndex..

[16] Mazarei Z, Rafati H (2019). Nanoemulsification of Satureja khuzestanica essential oil and pure carvacrol; comparison of physicochemical properties and antimicrobial activity against food pathogens.. LWT..

[17] Hong IK, Kim SI, Lee SB (2018). Effects of HLB value on oil-in-water emulsions: Droplet size, rheological behavior, zeta-potential, and creaming index.. J Industrial Engin Chem..

[18] Jung YT, Diosady LL (2012). Application of a Ternary Phase Diagram to the Phase Separation of Oil‐in‐Water Emulsions Using Isopropyl Alcohol.. J Am Oil Chemists' Soc..

[19] Silva-Flores PG, Perez-Lopez LA, Rivas-Galindo VM, Paniagua-Vega D, Galindo-Rodriguez SA, Alvarez-Roman R (2019). Simultaneous GC-FID Quantification of Main Components of Rosmarinus officinalis L. and Lavandula dentata Essential Oils in Polymeric Nanocapsules for Antioxidant Application.. J Anal Methods Chem..

[20] Bernardi DS, Pereira TA, Maciel NR, Bortoloto J, Viera GS, Oliveira GC (2011). Formation and stability of oil-in-water nanoemulsions containing rice bran oil: in vitro and in vivo assessments.. J Nanobiotechnol..

[21] Allende D, Cambiella A, Benito J M, Pazos C, Coca J (2008). Destabilization‐Enhanced Centrifugation of Metalworking Oil‐in‐Water Emulsions: Effect of Demulsifying Agents.. Chem Engin Technol..

[22] Latreille B, Paquin P (2006). Evaluation of Emulsion Stability by Centrifugation with Conductivity Measurements.. J Food Sci..

[23] Penjuri SCB, Damineni S, Ravouru N, Poreddy SR (2017). Self-Emulsifying Formulation of Indomethacin with Improved Dissolution and Oral Absorption.. Turk J Pharm Sci..

[24] Ameh SJ, Obodozie OO, Inyang US, Abubakar MS, Garba M, Preedy VR, Watson RR (2011). Climbing Black Pepper (Piper guineense) Seeds as an Antisickling Remedy.. Nuts and Seeds in Health and Disease Prevention..

[25] Ashokkumar K, Murugan M, Dhanya MK, Pandian A, Warkentin TD (2021). Phytochemistry and therapeutic potential of black pepper [Piper nigrum (L.)] essential oil and piperine: a review.. Clin Phytoscience..

[26] Chen S, Yang K, Xiang J, Raymond Kwaku O, Han J, Zhu X (2020). Comparison of Chemical Compositions of the Pepper EOs From Different Cultivars and Their AChE Inhibitory Activity.. Natural Product Communications..

[27] Mlost J, Kac P, Kedziora M, Starowicz K (2022). Antinociceptive and chondroprotective effects of prolonged beta-caryophyllene treatment in the animal model of osteoarthritis: Focus on tolerance development.. Neuropharmacology..

[28] Basholli-Salihu M, Schuster R, Hajdari A, Mulla D, Viernstein H, Mustafa B (2017). Phytochemical composition, anti-inflammatory activity and cytotoxic effects of essential oils from three Pinus spp.. Pharm Biol..

[29] Zhang L, Wu H, Li X, Zhang X, Li H, Tang H (2025). Screening bioactive compounds from Qianghuo (Notopterygium incisum) volatile oil via COX-2 magnetic ligand fishing.. Fitoterapia..

[30] Fraternale D, Teodori L, Rudov A, Prattichizzo F, Olivieri F, Guidarelli A (2018). The In Vitro Activity of Angelica archangelica L. Essential Oil on Inflammation.. J Med Food..

[31] Ocete MA, Risco S, Zarzuelo A, Jimenez J (1989). Pharmacological activity of the essential oil of Bupleurum gibraltaricum: anti-inflammatory activity and effects on isolated rat uteri.. J Ethnopharmacol..

[32] Jha NK, Sharma C, Hashiesh HM, Arunachalam S, Meeran MN, Javed H (2021). beta-Caryophyllene, A Natural Dietary CB2 Receptor Selective Cannabinoid can be a Candidate to Target the Trinity of Infection, Immunity, and Inflammation in COVID-19.. Front Pharmacol..

[33] Chen X, Ding Y, Guan H, Zhou C, He X, Shao Y (2024). The Pharmacological Effects and Potential Applications of Limonene From Citrus Plants: A Review.. Natural Product Communications..

[34] Khoshnazar M, Parvardeh S, Bigdeli MR (2020). Alpha-pinene exerts neuroprotective effects via anti-inflammatory and anti-apoptotic mechanisms in a rat model of focal cerebral ischemia-reperfusion.. J Stroke Cerebrovasc Dis..

